# MiR-3168, miR-6125, and miR-4718 as potential predictors of cisplatin-induced nephrotoxicity in patients with head and neck cancer

**DOI:** 10.1186/s12885-021-08317-2

**Published:** 2021-05-19

**Authors:** Julia C. F. Quintanilha, Maria A. Cursino, Jessica B. Borges, Nadine G. Torso, Larissa B. Bastos, Juliana M. Oliveira, Thiago S. Cobaxo, Eder C. Pincinato, Mario H. Hirata, Murilo V. Geraldo, Carmen S. P. Lima, Patricia Moriel

**Affiliations:** 1 School of Medical Science, University of Campinas, Campinas, São Paulo, Brazil; 2 Dante Pazzanese Institute of Cardiology, São Paulo, Brazil; 3 Faculty of Pharmaceutical Sciences, University of Campinas, 200 Cândido Portinari Street, Campinas, São Paulo 13083-871 Brazil; 4 Faculty of Pharmaceutical Sciences, University of São Paulo, São Paulo, Brazil; 5 Institute of Biology, University of Campinas, Campinas, São Paulo, Brazil

**Keywords:** Cisplatin, Nephrotoxicity, microRNAs, miR-3168, miR-6125, miR-4718

## Abstract

**Background:**

No biomarker is available for identifying cancer patients at risk of developing nephrotoxicity when treated with cisplatin.

**Methods:**

We performed microRNA (miRNA) sequencing using plasma collected 5 days after cisplatin treatment (D5) from twelve patients with head and neck cancer with and without nephrotoxicity (grade ≥ 2 increased serum creatinine). The most differentially expressed miRNAs between the two groups were selected for quantification at baseline and D5 in a larger cohort of patients. The association between miRNAs and nephrotoxicity was evaluated by calculating the odds ratio (OR) from univariate logistic regression. Receiver operating characteristic curves (ROC) were used to estimate the area under the curve (AUC), sensitivity, and specificity.

**Results:**

MiR-3168 (*p* = 1.98 × 10^− 8^), miR-4718 (*p* = 4.24 × 10^− 5^), and miR-6125 (*p* = 6.60 × 10^− 5^) were the most differentially expressed miRNAs and were further quantified in 43, 48, and 53 patients, respectively. The baseline expression of miR-3168 (*p* = 0.0456, OR = 1.03, 95% CI: 1.00–1.06) and miR-4718 (*p* = 0.0388, OR = 1.56, 95% CI: 1.03–2.46) were associated with an increased risk of nephrotoxicity, whereas miR-6125 showed a trend (*p* = 0.0618, OR = 1.73, 95% CI: 0.98–3.29). MiR-4718 showed the highest AUC (0.77, 95% CI: 0.61–0.93) with sensitivity of 66.76 and specificity of 79.49.

**Conclusions:**

We have provided evidence of baseline plasmatic expression of miR-3168, miR-6125, and miR-4718 as potential predictors of cisplatin-induced nephrotoxicity.

**Supplementary Information:**

The online version contains supplementary material available at 10.1186/s12885-021-08317-2.

## Background

Cisplatin is a widely used chemotherapeutic agent for treating several types of tumors, including head and neck cancer [1]. The antitumor efficacy of cisplatin relies on its interaction with DNA and the formation of DNA adducts through covalent bonds, mainly at N7 of adenine and guanine. The DNA adducts arrest the cell cycle in the G2 phase and block DNA replication, leading to the activation of apoptosis in tumor cells [2].

Despite the proven efficacy of cisplatin treatment, patients frequently experience toxicity that limits the duration and efficacy of therapy. Nephrotoxicity is one of the most frequent and dose-limiting cisplatin-induced toxicities, with a prevalence that varies between 30 and 50% in patients treated with ≥80 mg/m^2^ cisplatin [3, 4].

The mechanisms of cisplatin-induced nephrotoxicity are not entirely understood. The proposed mechanisms include the following: (1) activation of apoptosis due to cisplatin-induced damage in renal tubular cells [5]; (2) induction of oxidative stress resulting from increased production of reactive oxygen species due to cisplatin-induced damage in mitochondrial DNA [3]; (3) accumulation of cisplatin in renal tubular cells due to the high affinity of cisplatin to the copper transporter CTR1 and the organic cation transporter 2 (OCT2) [6, 7]; and (4) inhibition of Na^+^/K^+^ ATPase pump in renal cells by cisplatin [8].

Currently, no validated biomarkers are available for identifying patients at risk of cisplatin-induced nephrotoxicity and traditional markers used to evaluate nephrotoxicity after cisplatin administration, including serum creatinine (SCr) and blood urea nitrogen (BUN), lack specificity and sensitivity [9]. SCr is the most used indicator for detecting renal damage, but it is not an optimal renal maker, showing no significant changes in serum levels until approximately half of the nephrons are lost [10]. Thus, the need for identifying new biomarkers to evaluate cisplatin-induced nephrotoxicity.

Circulating microRNAs (miRNAs) have been investigated as potential biomarkers of cisplatin-induced nephrotoxicity because they are easily detected in biological fluids [11]. MiRNAs are small, single-stranded RNA molecules of approximately 22 non-coding nucleotides, which participate in the post-transcriptional regulation of gene expression [12]. Most studies were carried out using animal models [11]. Only one study was performed in urine samples of cisplatin-treated patients with mesothelioma to investigate specific miRNAs (miR-21, miR-200c, and miR-423) but could not validate a marker of cisplatin-induced nephrotoxicity [13]. Various circulating miRNAs are found in specific fluids; however, the same miRNAs can be found in different fluids. The plasma has the most variety of miRNAs, probably because it captures miRNAs from different cell types during circulation [14].

To our knowledge, this is the first study aimed to identify circulating plasma miRNAs as biomarkers of cisplatin-induced nephrotoxicity using the patients’ samples. We performed miRNA sequencing using plasma samples from patients with head and neck cancer, with and without cisplatin-induced nephrotoxicity, and tested three selected miRNAs for validation in a larger cohort of patients. Moreover, we performed a bioinformatics analysis to identify the mechanisms of miRNAs and their target genes to explain why only a subset of patients develop cisplatin-induced nephrotoxicity.

## Results

### Patient characteristics and toxicities

A total of 60 patients were included in this study. Nephrotoxicity (grade ≥ 2 increased SCr) was observed in 11 (18.3%) patients and grade 3 in 3 (5.0%) patients. No case of grade 4 increased SCr was reported.

Blood samples were collected before and 5 days after the first dose of cisplatin (D5) for SCr and BUN measurement. Plasma samples collected on D5 of 12 out of the 60 patients were selected for miRNAs sequencing and divided into two groups: samples of 6 patients with grade ≥ 2 increased SCr (nephrotoxicity group) and samples of 6 patients with grade = 0 increased SCr (non-nephrotoxicity group). The clinical characteristics and nephrotoxicity parameters of all patients included in the study and patients whose miRNA samples were sequenced are shown in Table 1.

**Table 1:** Patients with head and neck cancer treated with cisplatin. *KPS* Karnofsky performance status, *SD* standard deviation

Patient and clinical characteristics			
	Patients whose miRNA samples were sequenced		All patients (***n*** = 60)
	Non-nephrotoxicity group (***n*** = 6)	Nephrotoxicity group (***n*** = 6)	
**Age (mean ± SD, years)**	60.3 ± 4.9	57.5 ± 7.4	58.4 ± 7.3
**Gender (n, %)**			
Men	5 (83.3)	4 (66.7)	54 (90.0)
Women	1 (16.7)	2 (33.3)	6 (10.0)
**Ethnicity (n, %)**			
Caucasian	4 (66.7)	6 (100.0)	45 (75.0)
Non-Caucasian	2 (33.3)	0 (0.0)	15 (25.0)
**Smoking category (n, %)**			
Never smoked	1 (16.7)	1 (16.7)	6 (10.0)
Light smoker	0 (0.0)	0 (0.0)	3 (5.0)
Moderate smoker	0 (0.0)	1 (16.7)	4 (6.7)
Heavy smoker	5 (100.0)	4 (66.6)	47 (78.3)
**Drinking category (n, %)**			
Abstainer	2 (33.3)	2 (33.3)	9 (15.0)
Light drinker	0 (0.0)	0 (0.0)	5 (8.3)
Moderate drinker	0 (0.0)	0 (0.0)	2 (3.3)
Heavy drinker	3 (50.0)	1 (16.7)	16 (26.7)
Very heavy drinker	1 (16.7)	3 (50.0)	28 (46.7)
**KPS (n, %)**			
60	0 (0.0)	0 (0.0)	1 (1.7)
70	0 (0.0)	0 (0.0)	1 (1.7)
80	2 (33.3)	3 (50.0)	9 (15.0)
90	4 (66.7)	3 (50.0)	40 (66.6)
100	0 (0.0)	0 (0.0)	9 (15.0)
**Tumor site (n, %)**			
Oropharynx	0 (0.0)	1 (16.7)	15 (25.0)
Hypopharynx	2 (33.3)	3 (50.0)	9 (15.0)
Larynx	1 (16.7)	1 (16.7)	13 (21.7)
Oral cavity	3 (50.0)	1 (16.7)	20 (33.3)
Hypopharynx + Larynx	0 (0.0)	0 (0.0)	1 (1.7)
na	0 (0.0)	0 (0.0)	2 (3.3)
**Stage (n, %)**			
I	0 (0.0)	0 (0.0)	0 (0.0)
II	0 (0.0)	0 (0.0)	6 (10.0)
III	1 (16.7)	0 (0.0)	7 (11.6)
IV	5 (83.3)	6 (100.0)	46 (76.7)
na	0 (0.0)	0 (0.0)	1 (1.7)
**Nephrotoxicity parameters**			
**Serum creatinine, SCr (mg/dL)**			
Baseline	0.94 ± 0.18	0.75 ± 0.10	0.82 ± 0.20
D5	0.94 ± 0.21	3.39 ± 2.13	1.36 ± 1.01
**Creatinine clearance (mL/min)**			
Baseline	70.20 ± 22.93	97.62 ± 40.55	87.70 ± 26.90
D5	69.72 ± 24.01	25.05 ± 11.46	61.86 ± 25.54
**Blood urea nitrogen, BUN (mg/dL)**			
Baseline	38.50 ± 14.28	21.33 ± 9.48	28.75 ± 10.95
D5	45.67 ± 14.88	92.00 ± 40.61	53.63 ± 22.99

### MiRNA sequencing results

Thirty-three miRNAs were differentially expressed between the nephrotoxicity and non-nephrotoxicity groups with a fold regulation (FR) > 2 or FR < − 2, with no comments or comment “A” according to the GeneGlobe Data Analysis Center (Qiagen) (Supplementary Table S1). The most differentially expressed miRNAs were miR-3168 (*p* = 1.98 × 10^− 8^, FR = 8.08), miR-6125 (*p* = 6.60 × 10^− 5^, FR = 5.31), and miR-4718 (*p* = 4.24 × 10^− 5^, FR = 5.12) upregulated in the nephrotoxicity group. Volcano plots for all miRNAs detected through sequencing (including those with comments “B” and “C”) are shown in Supplementary Fig. S1.

### Validation of miR-3168, miR-6125, and miR-4718

The expression of miR-3168, miR-6125, and miR-4718 was verified in 50, 60, and 55 patients, respectively. However, 7 patients were excluded because cel-miR-39 expression was higher than 2 standard deviations (SD, 2 patients with grade ≥ 2 increased SCr and 5 patients with grade < 2 increased SCr). Thus, 43, 53, and 48 patients were analyzed for the expressions of miR-3168, miR-6125, and miR-4718, respectively.

**Fig. 1:**
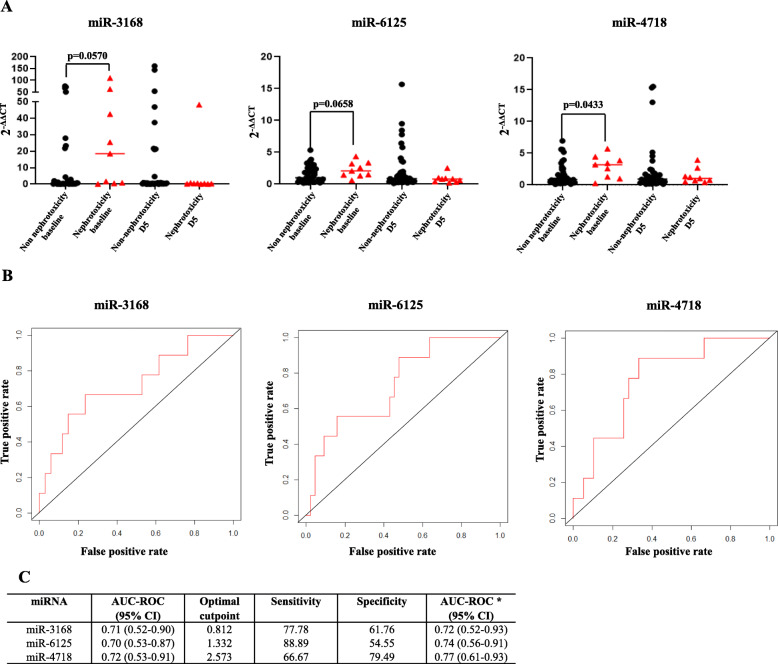
Expression and predictive performance of miR-3168, miR-6125, and miR-4718.** a.** Expression of miR-3168, miR-6125, and miR-4718 in the nephrotoxicity and non-nephrotoxicity group before and five days (D5) after cisplatin administration. **b**. Receiver operating characteristic curve (ROC) of baseline miRNAs expression adjusted for age and gender. **c**. Details of the area under the curve (AUC), cutoff, sensitivity, and specificity of miRNAs expression before cisplatin administration as prognostic markers of cisplatin-induced nephrotoxicity

MiR-4718 expression was higher before cisplatin administration in patients with grade ≥ 2 increased SCr (*p* = 0.0433). MiR-3168 (*p* = 0.0570) and miR-6125 (*p* = 0.0658) also showed a trend toward higher expression in baseline samples of patients with grade ≥ 2 increased SCr (Fig. 1 and Supplementary Table S2). Thus, we focused our further analyses on evaluating the baseline expressions of miR-3168, miR-6125, and miR-4718.

MiR-4718 (*p* = 0.0388, odds ratio, OR = 1.56, 95% confidence interval, CI: 1.03–2.46) and miR-3168 (*p* = 0.0456, OR = 1.03, 95% CI: 1.00–1.06) were statistically significantly associated with a higher risk of grade ≥ 2 increased SCr and miR-6128 showed a trend (*p* = 0.0681, OR = 1.73, 95% CI: 0.98–3.29) in the univariate regression analyses. In the predictive performance analysis (Fig. 1), miR-4718 showed the highest area under the curve (AUC = 0.77, 95% CI: 0.61–0.93) and specificity (79.49), whereas miR-6125 showed the highest sensitivity (88.89).

The baseline expressions of miR-3168, miR-6125, and miR-4718 were highly correlated (Supplementary Table S3), with a variance inflation factor (VIF) of 3.60, 3.82, and 4.37, respectively, indicating moderate multicollinearity. In multivariate logistic regression analysis (Supplementary Table S4), none of the miRNAs were statistically significantly associated with grade ≥ 2 increased SCr, probably due to multicollinearity. We combined baseline miRNA expression by summing the expression values of miR-3168, miR-6125, and miR-4718 and performed an univariate logistic regression, adjusting for age and gender. Although the combined miRNAs were statistically significantly associated with a higher risk of grade ≥2 increased SCr (*p* = 0.0409, OR = 1.03, 95% CI: 1.00–1.05) with AUC = 0.73 (95% CI: 0.51–0.93), no advantage in prediction performance was observed compared with individually analyzed miRNAs.

### Bioinformatics analysis

Differentially expressed miRNAs, with an FR > 2.5 (upregulated), including miR-3168, miR-6125, miR-4718, miR-5694, miR-203a-3p, miR-141-5p, miR-7977, and miR-1303, were selected for bioinformatics analysis. The matrix, showing the top 100 target genes predicted using the upregulated miRNAs is shown in Supplementary Fig. S2. MiR-7977 and miR-203a-3p did not present targets predicted using at least six algorithms and were excluded from further analysis.

Differentially expressed miRNAs with an FR < − 2.5 (downregulated), including miR-17-5p, miR-1185-1-3p, miR-766-3p, miR-151b, miR-151a-5p, and miR-485-3p, were selected for bioinformatics analysis. The matrix with the top 100 predicted target genes of downregulated miRNAs is shown in Supplementary Fig. S3.

**Fig. 2:**
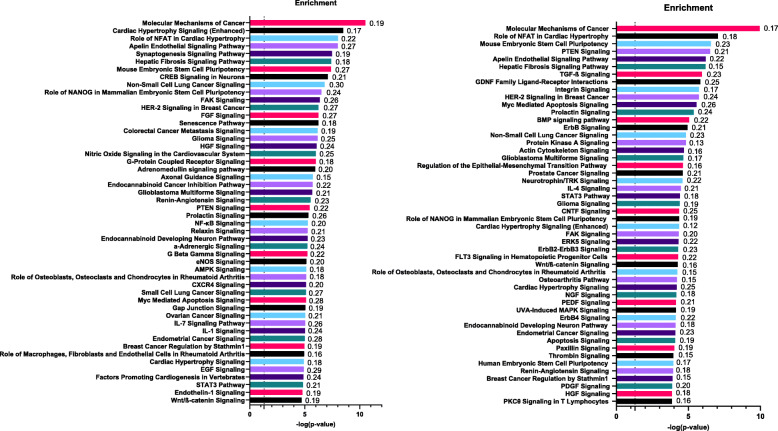
Enrichment analysis (top 50 canonical signaling pathways) of the predicted target genes by up (left) and down (right) regulated plasmatic miRNAs. Enrichment analysis performed by Ingenuity Pathway Analysis (IPA®, Qiagen bioinformatics). The dashed line represents –log (*p*-value) = 1.3 or *p*-value = 0.05 (Fisher’s exact test). The ratios between the target miRNA genes and all genes involved in the specific signaling pathway are represented on the right side of each bar

Gene set enrichment analysis was performed with 2017 predicted target genes of upregulated miRNAs and 1677 predicted target genes of downregulated miRNAs. The top 50 canonical signaling pathways of predicted target genes of upregulated and downregulated miRNAs are shown in Fig. 2. For both upregulated and downregulated miRNAs, an enrichment of signaling pathways involved in cisplatin-induced nephrotoxicity was observed, including the nuclear factor kappa B (NF-κB), AMP-activated protein kinase (AMPK), IL-1 signaling pathways, transforming growth factor β (TGF-β), and ErbB/ErbB2-ErbB3/ErbB4 signaling pathways.

**Fig. 3:**
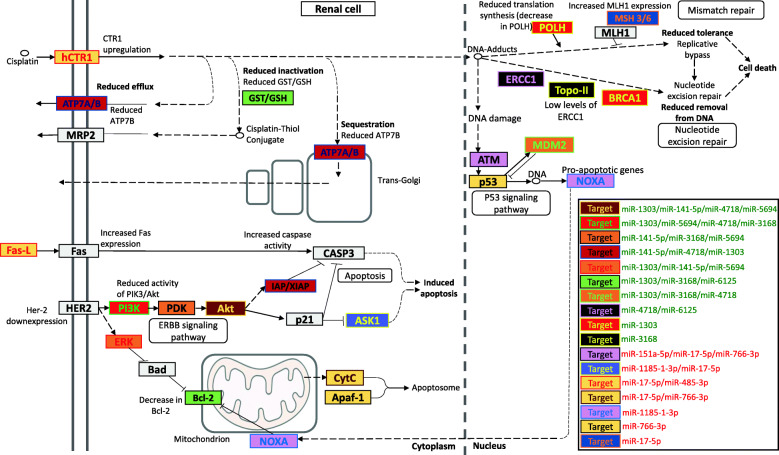
Predicted target genes of plasmatic miRNAs differentially expressed in patients with cisplatin-induced nephrotoxicity. The miRNAs in green are upregulated whereas those in red are downregulated. Color combinations are used for target genes to differentiate between genes according to regulatory miRNAs

Figure 3 highlights the target genes from the miRNA matrices directly involved in cisplatin-induced nephrotoxicity, which can partially explain the nephrotoxicity in patients from the nephrotoxicity group.

## Discussion

We have investigated, for the first time, the role of plasma miRNAs as biomarkers of cisplatin-induced nephrotoxicity using patients’ samples. We used miRNA sequencing to identify miR-3168, miR-6125, and miR-4718 as potential biomarkers of cisplatin-induced nephrotoxicity. The evaluation of miRNAs in a larger cohort of patients provides more evidence of their potential utility. A bioinformatics analysis approach was used to build the post-transcriptional network regulated by the miRNAs and identify genes affected by the differential expression of miRNAs and explain why a subset of cancer patients develop nephrotoxicity during cisplatin treatment.

We performed a miRNA sequencing of plasma samples collected from six patients with and without nephrotoxicity on D5, respectively. The most statistically significant differentially expressed miRNAs (miR-3168, miR-6125, and miR-4718) between the two groups of patients were quantified in the plasma samples of a larger cohort of patients. Interestingly, miR-3168, miR-6125, and miR-4718 had a higher expression in baseline samples of patients who developed nephrotoxicity after cisplatin treatment (miR-4718 was statistically significant, whereas miR-3168 and miR-6125 showed a trend) and we focused our analysis on their utility as predictors of cisplatin-induced nephrotoxicity.

MiR-4718 was found to be the most promising marker associated with grade ≥ 2 increased SCr, with an AUC of 77, sensitivity of 66.76 and specificity of 79.49 in predicting cisplatin-induced nephrotoxicity (Fig. 1). MiR-3168 was also statistically significant associated with grade ≥ 2 increased SCr; however, it showed the lowest predictive performance. MiR-6125 did not reach statistical significance, with only a trend being observed, but showed an intermediate predictive performance when compared to miR-4718 and miR-3168 and had the best sensitivity (88.89) in predicting cisplatin-induced nephrotoxicity. MiR-3168, miR-6125, and miR-4718 correlated with each other, and multicollinearity does not suggest an additive effect of the miRNAs in a multivariate model. Thus, the potential of miR-3168, miR-6125, and miR-4718 as predictors of cisplatin-induced nephrotoxicity might rely on their individual effects.

To understand how miR-3168, miR-6125, and miR-4718 are involved in cisplatin-induced nephrotoxicity, we used a bioinformatics approach for the three candidate miRNAs and additional miRNAs with FR > 2.5 or FR < − 2.5 identified through sequencing in this study. Here, we make some claims based on the bioinformatics analysis that should be further validated in functional experiments/analyses. Target gene enrichment analysis revealed critical signaling pathways involved in cisplatin-induced nephrotoxicity, including the ErbB, NF-κB, TGF-β, IL-1, and AMPK pathways (Fig. 2). Upregulated miRNAs targeting genes of the ErbB signaling pathway, such as miR-3168 (*p* = 1.98 × 10^− 8^ FR = 8.08), miR-5694 (*p* = 0.0002, FR = 4.13), and miR-141-5p (*p* = 0.0033, FR = 2.95), which target *PDK* according to our bioinformatics analysis, could downregulate the pathway, leading to cisplatin-induced apoptosis in renal cells (Fig. 3). MiR-146b was shown to contribute to nephrotoxicity by reducing ErbB4/HER4 expression [15]; although miR-146b was not identified in our study, this corroborates our hypothesis that miRNAs are key regulators of the ErbB signaling pathway during cisplatin-induced nephrotoxicity.

The NF-κB signaling pathway is involved in the induction of miR-375 expression by cisplatin, suppressing the activation of hepatocyte nuclear factor-1β (HNF-1β), which acts as a nephroprotectant and prevents renal damage in tubular cells [16]. Cisplatin has been reported to induce NF-κB phosphorylation and its subsequent translocation to the nucleus, promoting the transcription of inflammatory factors, including TNF-α and IL-1, which contribute to renal damage [17, 18]. TNF-α plays a central role in the inflammatory response triggered by cisplatin in the renal cell [19] and contributes to a significant increase in the expression of IL-1β and TGF-β mRNA in rat renal cells after cisplatin treatment [20]. IL-1β is also involved in the recruitment of monocytes in the inflamed renal parenchyma [17].

AMPK regulates autophagy in kidney cells. The inhibition of AMPK was reported to suppress cisplatin-induced autophagy in tubular cells, followed by increased DNA damage and activation of p53 [21]. Autophagy contributes toward removing damaged mitochondria and decrease oxidative stress [22]. The inhibition of autophagy in renal cells may also be related to increased oxidative stress, hence, increased nephrotoxicity. Several upregulated miRNAs identified in patients with nephrotoxicity in our study (including miR-4718, *p* = 4.24 × 10^− 5^, FR = 5.12, and miR-3168, *p* = 1.98 × 10^− 8^, FR = 8.08) target members of the AMPK signaling pathway, mainly *PRKAB2* (Supplementary Fig. S2), which could explain nephrotoxicity by repression of autophagy in tubular cells.

This study also aimed to explore where the differentially expressed miRNAs act during the mechanism of action of cisplatin inside the renal cell (Fig. 3). Important genes involved in the mechanism of action of cisplatin are potentially targeted by the miRNAs identified in our sequencing. These differentially expressed miRNAs probably co-operate to modulate different pathways and could be released into the plasma upon renal damage and detected as circulating miRNAs.

The ErbB signaling pathway is an example of the contribution of different miRNAs in regulating genes that encode proteins that interact and lead to cisplatin-induced apoptosis in renal cells, as shown in Fig. 3. The DNA repair pathway is another example. The reducing activity of the nucleotide-excision repair (NER) pathway could reduce the cell’s ability to repair cisplatin-induced DNA damage, thereby inducing cell death [23], due to the downregulation of *ERCC1* (by miR-4718, *p* = 4.24 × 10^− 5^ FR = 5.12, and miR-6125, *p* = 6.60 × 10^− 5^ FR = 5.31), *TOP2A* (by miR-3168, *p* = 1.98 × 10^− 8^ FR = 8.08), and *BRCA1* (by miR-1303, *p* = 0.0201 FR = 2.57).

The downregulation of miR-17-5p (*p* = 2.49 × 10^− 5^, FR = − 3.42) and miR-485-3p (*p* = 0.0073, FR = − 2.56) could explain the increased activity of cisplatin receptor CTR1 (Fig. 3), which leads to a higher uptake of cisplatin by renal cells, leading to nephrotoxicity [7]. The regulation of genes involved in the mitochondrial apoptosis pathway may also contribute to higher nephrotoxicity, suggested by a decrease in the activity of the anti-apoptotic protein Bcl-2 by miR-3168 (*p* = 1.98 × 10^− 8^, FR = 8.08), miR-6125 (*p* = 6.60 × 10^− 5^, FR = 5.31), and miR-1303 (*p* = 0.201, FR = 2.57) (Fig. 3). In addition, genes of the cisplatin detoxification pathway, which includes the conjugation of cisplatin with glutathione, are also shown to be target of miR-3168, miR-6125 and miR-1303 (Fig. 3), which could reduce the content of glutathione S-transferase (GST) and reduced glutathione (GSH). Although the role of GST in cisplatin-induced nephrotoxicity is controversial [24], evidence suggests that the decreased GST/GSH leads to a higher concentration of nonconjugated cisplatin inside the renal cell, contributing to higher nephrotoxicity [25].

There are some limitations in the analyses presented. Our study comprised of 60 patients, of which 11 experienced grade ≥ 2 increased SCr; thus, the sample size was small, which can generate bias in the estimated AUC. Furthermore, given the number of events and concerns of overfitting, we adjusted the analysis only for age and gender. Potential confounders might also be considered, such as concomitant medication use, smoking, and pre-existing conditions. The impact of these confounding variables thus remains a question for future studies. We have performed only in silico analysis to hypothesize how miRNAs can modulate cisplatin-induced nephrotoxicity and our hypotheses should be evaluated through functional experiments/analyses. Moreover, our findings require independent confirmation in a larger cohort of patients.

## Conclusions

In conclusion, this study provided evidence of baseline plasmatic expression of miR-3168, miR-6125, and miR-4718 as potential predictors of cisplatin-induced nephrotoxicity. This is the first evidence of plasmatic miRNAS quantified human samples showing potential biomarker utility, and its implementation to the clinical practice should be further assessed in pharmacoeconomic studies. The expression of miR-3168, miR-6125, and miR-4718 could be evaluated by a simple qPCR before treatment to improve the use of cisplatin in the clinical setting and selecting patients to be treated with cisplatin with an improved risk/benefit ratio.

## Methods

### Patient selection, treatment regimen, and toxicity

This is a nested case-control study. The inclusion criteria consisted of outpatients between 18 and 80 years old with primary squamous cell carcinoma of the head and neck (oral cavity, oropharynx, hypopharynx, and larynx) who received one cycle of high doses of cisplatin (80 or 100 mg/m^2^) concomitantly with conventional radiotherapy. Patients were excluded if a second primary tumor was present, they declined to participate at any time during the course of the study, or they did not provide blood samples for the study.

Blood samples were collected before cisplatin administration and on D5 for SCr and BUN measurement. SCr clearance was estimated using the Cockcroft–Gault formula [26]. Nephrotoxicity was classified as grade ≥ 2 increased SCr according to Common Toxicity Criteria for Adverse Events (CTCAE) version 4. Grade ≥ 2 increased SCr is classified as more than two times SCr baseline values.

### Sequencing of miRNAs

MiRNAs were extracted from samples collected on D5 from six patients with grade ≥ 2 increased SCr (nephrotoxicity group) and six patients with grade = 0 increased SCr (non-nephrotoxicity group) using the miRNeasy Serum/Plasma Kit (Qiagen, cat no. 217184). Library preparation was performed using the QIAseq™ miRNA Library Kit (Qiagen, cat no. 331502). The quality control of the libraries was performed by analyzing their size using the 4200 TapeStation Instrument (Agilent Technologies) and the concentration by using Qubit Fluorometric Quantitation (Thermo Fisher Scientific). The molarity of each library was calculated and diluted to 4 nM for sequencing.

Sequencing was performed using the MiSeq Reagent Kit v3, 150-cycle (Illumina, MS-102-3001). Sequencing data analysis was performed using the GeneGlobe Data Analysis Center (Qiagen). DESeq2 method was used for normalization. GeneGlobe provides the fold-change (FC = miRNA expression of the nephrotoxicity group / miRNA expression of the non-nephrotoxicity group), FR (FR = FC, if FC ≥1 or FR = 1/FC, if FC < 1) and *p*-value based on the Wald test for each miRNA. GeneGlobe also provides comments for different miRNAs. These comments can be “A”, if the miRNA’s average expression level is relatively low (< 10) in either the nephrotoxicity or the non-nephrotoxicity group and is reasonably high in the other group (> 10); “B”, if the miRNA’s average expression level is relatively low (< 10) in both the nephrotoxicity and non-nephrotoxicity groups; or “C”, if the miRNA has an expression count of zero in both the nephrotoxicity and non-nephrotoxicity groups, meaning that its expression was undetected, making the FC result erroneous and uninterpretable.

### Validation of selected miRNAs

The three most statistically significant differentially expressed miRNAs, with the highest FR identified through sequencing, were selected for validation in a larger cohort of patients. After extracting the miRNAs from all plasma samples collected on the baseline and on D5 from all patients included in the study, cDNA synthesis was performed using the TaqMan™ Advanced miRNA cDNA Synthesis Kit (Applied Biosystems, Cat No./ID: A28007) and qPCR using TaqMan™ Advanced miRNA Assays (Applied Biosystems, Cat No./ID:A25576). In addition, qPCR of the exogenous control cel-miR-39 and the endogenous control hsa-miR-16 for normalization was performed. Hsa-miR-16 was selected as an endogenous control because its expression was shown to be stable in plasma samples [27]. We normalized our analyses using cel-miR-39 due to its smaller variations of cycle threshold (CT) than has-miR-16. Patients with cel-miR-39 expression above two SDs were excluded from the analysis.

MiRNA qPCR results were analyzed using the QuantStudioTM Real Time PCR Software 6. Each miRNA had its expression evaluated and relative expressions were obtained using the 2^−∆∆CT^ method [28], where ∆CT = candidate CTmiRNA − CT cel-miR-39 and ∆∆CT = ∆CT – mean ∆CTs of patients with grade < 2 increased SCr.

### Statistical analysis

Statistical analysis was performed using the R software environment for statistical computing [29]. MiRNA expression was compared between the nephrotoxicity and non-nephrotoxicity groups at baseline and D5 using the Mann–Whitney U tests. For comparisons with a *p*-value of < 0.10, we tested the association between miRNA expression and grade ≥ 2 increased SCr by calculating the OR from a univariate logistic regression analysis, considering grade ≥ 2 increased SCr as a binary outcome, adjusted for age and gender. Receiver operating characteristic curves (ROC) were used to estimate the AUC, optimal cutpoint, sensitivity, and specificity.

Spearman correlations and multicollinearity using VIF were calculated for miRNA expression. Multivariate logistic regression analyses were performed to evaluate the relationship between grade ≥ 2 increased SCr and miRNAs expression at baseline. Age and gender were included as covariates in the multivariate model.

### Bioinformatics analysis

Differentially expressed miRNAs with FR > 2.5 or FR < − 2.5 were selected for bioinformatics analysis. All miRNAs selected presented no comments or comment “A” and a *p*-value of < 0.05. The identification of predicted miRNA target genes was performed using the miRWalk platform [30], which provides a list of predicted miRNA target genes according to twelve different algorithms, including TargetScan [31]. Only target genes predicted by TargetScan and at least five different databases were selected for the following analyses.

Two matrices were constructed to identify the interaction between miRNAs and their predicted target genes: one for upregulated miRNAs and one for downregulated miRNAs. The matrices were sorted according to the potential target genes of different miRNAs (stronger evidence); genes targeted by at least two different miRNAs were selected for unsupervised enrichment analysis using the Ingenuity Pathway Analysis (IPA®, Qiagen) software to identify the main canonical signaling pathways involving differentially expressed miRNAs.

The KeggMapper [32] was used to visualize the canonical signaling pathways involving miRNA target genes. We selected the signaling pathway for platinum-resistance and adapted it to the signaling pathway of cisplatin-induced nephrotoxicity in renal cells. Thus, we could visualize how miRNAs regulate genes in cisplatin-induced nephrotoxicity.

## Supplementary Information

**Additional file 1: Table S1**. Plasmatic miRNAs with a *p*-value < 0.05, FR > 2.0 or FR < -2.0, without comments or with comment “A” according to the GeneGlobe Data Analysis Center (Qiagen). **Table S2**. MiRNA expression at baseline and D5. **Table S3**. Spearman correlation for miR-3168, miR-4718, and miR-6125. **Table S4**. Multivariate logistic regression between miRNAs and grade ≥ 2 increase in serum creatinine, using age and gender as covariates. **Fig. S1.** Volcano plot of miRNA sequencing. **Fig. S2.** Top 100 target genes predicted by upregulated miRNAs according to miRWalk 2.0. **Fig. S3**. Top 100 target genes predicted by downregulated miRNAs according to miRWalk 2.0.

## Data Availability

The datasets generated and/or analyzed during the current study are available in the Research data repository of the University of Campinas, https://redu.unicamp.br/dataset.xhtml?persistentId=doi:10.25824/redu/XT1BEY
