# Supplementary material for: MiR-3168, miR-6125, and miR-4718 as potential predictors of cisplatin-induced nephrotoxicity in patients with head and neck cancer
Source: BMC Cancer. 2021 May 19;21:575. doi: 10.1186/s12885-021-08317-2 (PMC8136168; doi:10.1186/s12885-021-08317-2)
Supplement: Supplementary file 1 — Additional file 1: Table S1. Plasmatic miRNAs with a p-value < 0.05, FR > 2.0 or FR < -2.0, without comments or with comment “A” according to the GeneGlobe Data Analysis Center (Qiagen). Table S2. MiRNA expression at baseline and D5. Table S3. Spearman correlation for miR-3168, miR-4718, and miR-6125. Table S4. Multivariate logistic regression between miRNAs and grade ≥ 2 increase in serum creatinine, using age and gender as covariates. Fig. S1. Volcano plot of miRNA sequencing. Fig. S2. Top 100 target genes predicted by upregulated miRNAs according to miRWalk 2.0. Fig. S3. Top 100 target genes predicted by downregulated miRNAs according to miRWalk 2.0. [file 12885_2021_8317_MOESM1_ESM.docx]

**MiR-3168, miR-6125, and miR-4718 as potential predictors of cisplatin-induced nephrotoxicity in patients with head and neck cancer**

**Running title:** MiRNAs predictors of cisplatin-induced nephrotoxicity

Julia C.F. Quintanilha^1^, Maria A. Cursino^1^, Jessica B. Borges^2^, Nadine G. Torso^3^, Larissa B. Bastos^3^, Juliana M. Oliveira^3^, Thiago S. Cobaxo^3^, Eder Pincinato^1^, Mario H. Hirata^2,4^, Murilo V. Geraldo^5^, Carmen S.P. Lima^1^, Patricia Moriel^1,3*^

^1^School of Medical Science, University of Campinas, Campinas, São Paulo, Brazil.

^2^Dante Pazzanese Institute of Cardiology, São Paulo, São Paulo, Brazil.

^3^Faculty of Pharmaceutical Sciences, University of Campinas, Campinas, São Paulo, Brazil.

^4^Faculty of Pharmaceutical Sciences, University of São Paulo, São Paulo, São Paulo, Brazil.

^5^Institute of Biology, University of Campinas, Campinas, São Paulo, Brazil.

^*^Correspondence: Patricia Moriel, PhD. Faculty of Pharmaceutical Sciences, University of Campinas, 200 Cândido Portinari Street, Campinas 13083-871, SP, Brazil. E-mail: patricia.moriel@fcf.unicamp.br

**Supplementary File**

**Tables and Figures**

**Table S1.** **Plasmatic miRNAs with a *p*-value <0.05, FR >5.0 or FR <-2.5, without comments or with comment “A” according to the GeneGlobe Data Analysis Center (Qiagen).** *FR* Fold Regulation, ^*^Wald test.

| **Plasmatic miRNAs** | | | | | | | |
| --- | --- | --- | --- | --- | --- | --- | --- |
| **miRNA** | **FR** | **p-value^*^** | **Comment** | **miRNA** | **FR** | **p-value*** | **Comment** |
| **hsa-miR-3168** | **8.08** | **1.98x10^-8^** | **-** | hsa-miR-17-5p | -3.42 | 2.49x10^-5^ | - |
| **hsa-miR-6125** | **5.31** | **6.60x10^-5^** | **-** | hsa-miR-1185-1-3p | -3.03 | 0.0384 | A |
| **hsa-miR-4718** | **5.12** | **4.24x10^-5^** | **-** | hsa-miR-766-3p | -2.77 | 0.0325 | A |
| hsa-miR-5694 | 4.13 | 0.0002 | A | hsa-miR-151b/151a-5p | -2.63 | 0.0046 | - |
| hsa-miR-203a-3p | 3.71 | 0.0037 | A | hsa-miR-485-3p | -2.56 | 0.0073 | A |
| hsa-miR-141-5p | 2.95 | 0.0033 | - | hsa-miR-652-3p | -2.45 | 0.0161 | A |
| hsa-miR-7977 | 2.70 | 0.0172 | A | hsa-miR-27a-3p | -2.44 | 0.0076 | - |
| hsa-miR-1303 | 2.57 | 0.0201 | A | hsa-miR-342-3p | -2.30 | 0.0010 | - |
| hsa-miR-944 | 2.40 | 0.0459 | - | hsa-miR-223-3p | -2.26 | 0.0017 | - |
| hsa-miR-9-5p | 2.35 | 0.0253 | A | hsa-miR-130a-3p | -2.20 | 0.0240 | - |
| hsa-miR-625-3p | 2.33 | 0.0175 | - | hsa-miR-127-3p | -2.19 | 0.0237 | - |
| hsa-miR-4782-5p | 2.21 | 0.0457 | - | hsa-miR-409-3p | -2.17 | 0.0094 | - |
| hsa-miR-6857-3p | 2.21 | 0.0461 | A | hsa-miR-24-3p | -2.15 | 0.0060 | - |
| hsa-miR-1224-5p | 2.13 | 0.0388 | A | hsa-miR-103a-3p | -2.14 | 0.0227 | - |
| hsa-miR-363-5p | 2.12 | 0.0455 | - | hsa-miR-23a-3p | -2.11 | 0.0155 | - |
| hsa-miR-6728-5p | 2.04 | 0.0381 | - | hsa-miR-423-3p | -2.05 | 0.0100 | - |
|  |  |  |  | hsa-miR-182-5p | -2.00 | 0.0252 | - |

**Table S2. MiRNA expression at baseline and D5.** *SCr* serum creatinine, *FC* fold change, *Mann–Whitney U test.

| **miRNA** | **Patients with grade <2 increased SCr Baseline** | **Patients with grade ≥2 increased SCr Baseline** | **FC** | **p-value^*^** |
| --- | --- | --- | --- | --- |
| miR-3168 | 10.54 ± 22.08 | 29.09 ± 37.31 | 2.76 | 0.0570 |
| miR-6125 | 1.50 ± 1.24 | 2.27 ± 1.19 | 1.51 | 0.0658 |
| miR-4718 | 1.66 ± 1.75 | 2.86 ± 1.76 | 1.72 | 0.0433 |
| **miRNA** | **Patients with grade <2 increased SCr**  **D5** | **Patients with grade ≥2 increased SCr**  **D5** | **FC** | **p-value^*^** |
| miR-3168 | 15.34 ± 37.51 | 5.66 ± 15.93 | 0.37 | 0.3689 |
| miR-6125 | 2.09 ± 3.13 | 0.84 ± 0.89 | 0.40 | 0.3555 |
| miR-4718 | 2.24 ± 3.81 | 1.40 ± 1.17 | 0.62 | 0.7554 |

**Table S3. Spearman correlation for miR-3168, miR-4718, and miR-6125.**

| **miRNA1** | **miRNA2** | **R** | **p-value** |
| --- | --- | --- | --- |
| miR-3168 | miR-6125 | 0.82 | 1.96x10^-11^ |
| miR-3168 | miR-4718 | 0.87 | 3.80 x10^-14^ |
| miR-6125 | miR-4718 | 0.79 | 2.13x10^-11^ |

**Table S4. Multivariate logistic regression between miRNAs and grade ≥2 increase in serum creatinine, using age and gender as covariates.** *OR* odds ratio, *CI* confidence interval.

|  | **p-value** | **OR (95% CI)** |
| --- | --- | --- |
| miR-3168 | 0.7360 | 1.01 (0.96-1.06) |
| miR-6125 | 0.9740 | 1.02 (0.31-2.95) |
| miR-4718 | 0.3900 | 1.46 (0.62-3.66) |
| Age | 0.5320 | 1.04 (0.92-1.19) |
| Gender | 0.1400 | 5.27 (0.52-53.10) |


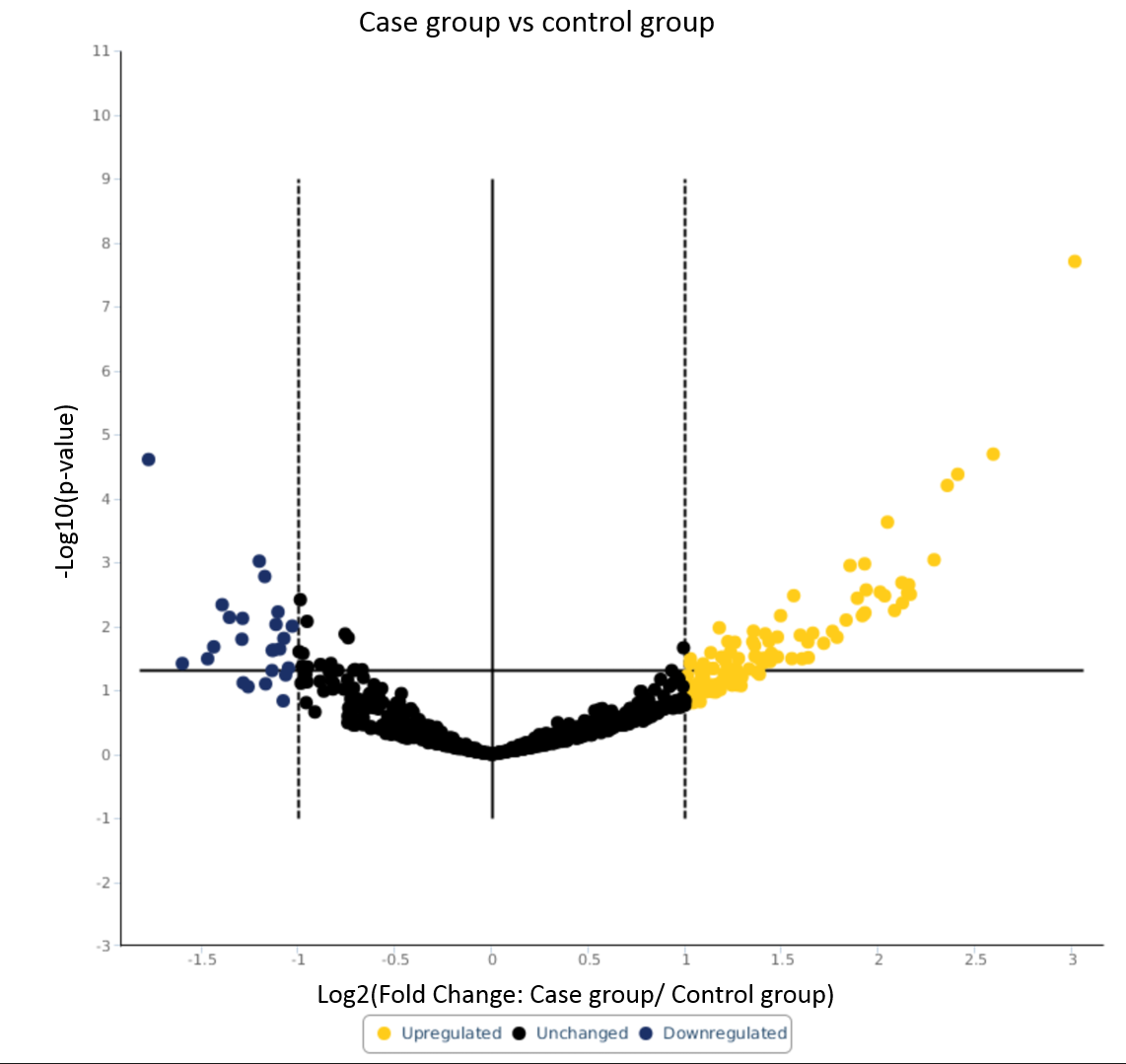


**Figure S1. Volcano plot of miRNA sequencing.**

**
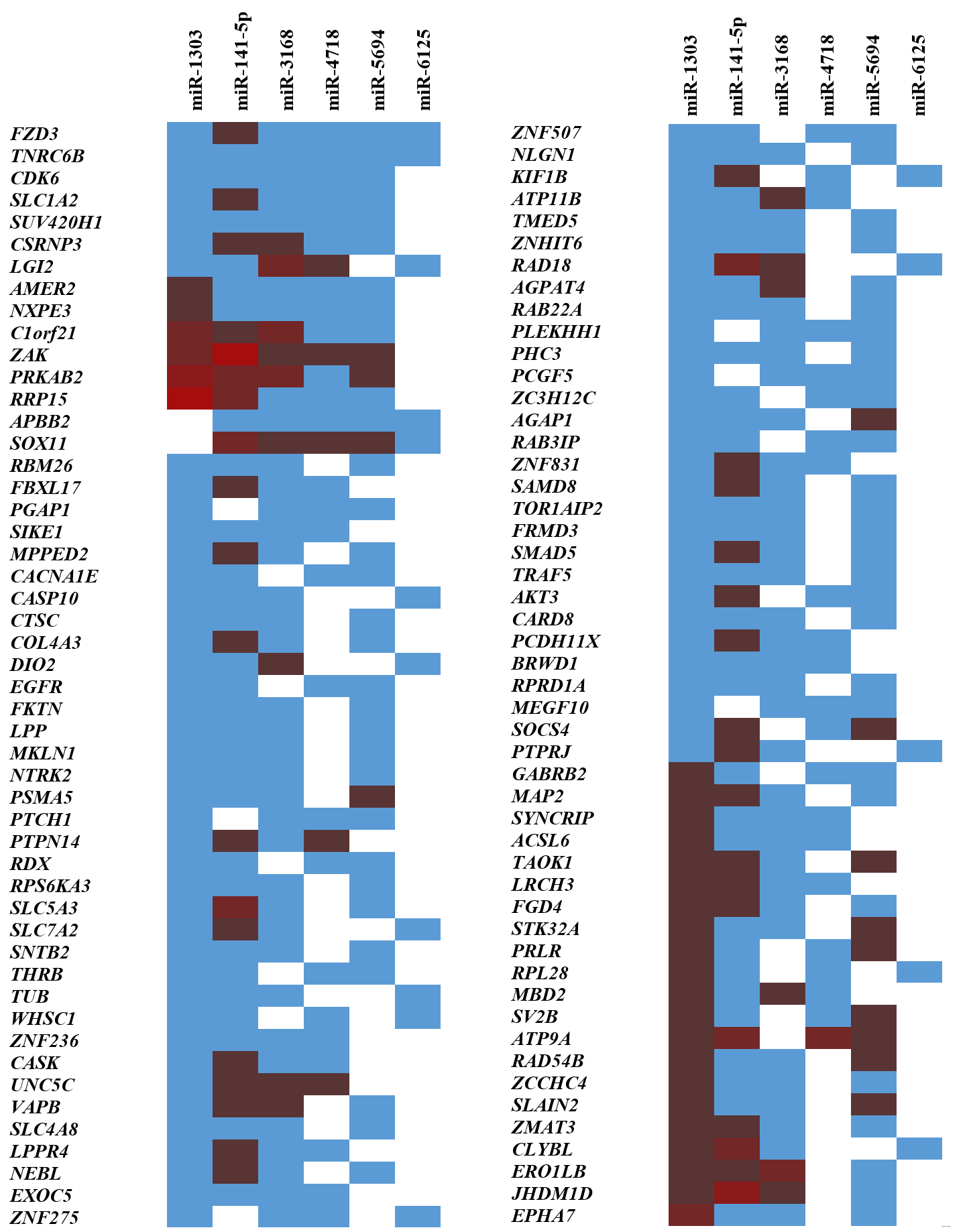
**
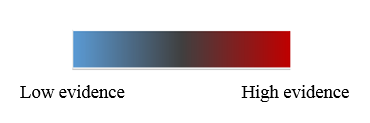


**Figure S2. Top 100 target genes predicted by upregulated miRNAs according to miRWalk 2.0.** The top genes are the targets predicted by more miRNAs and the color represents the evidence of this prediction.

*
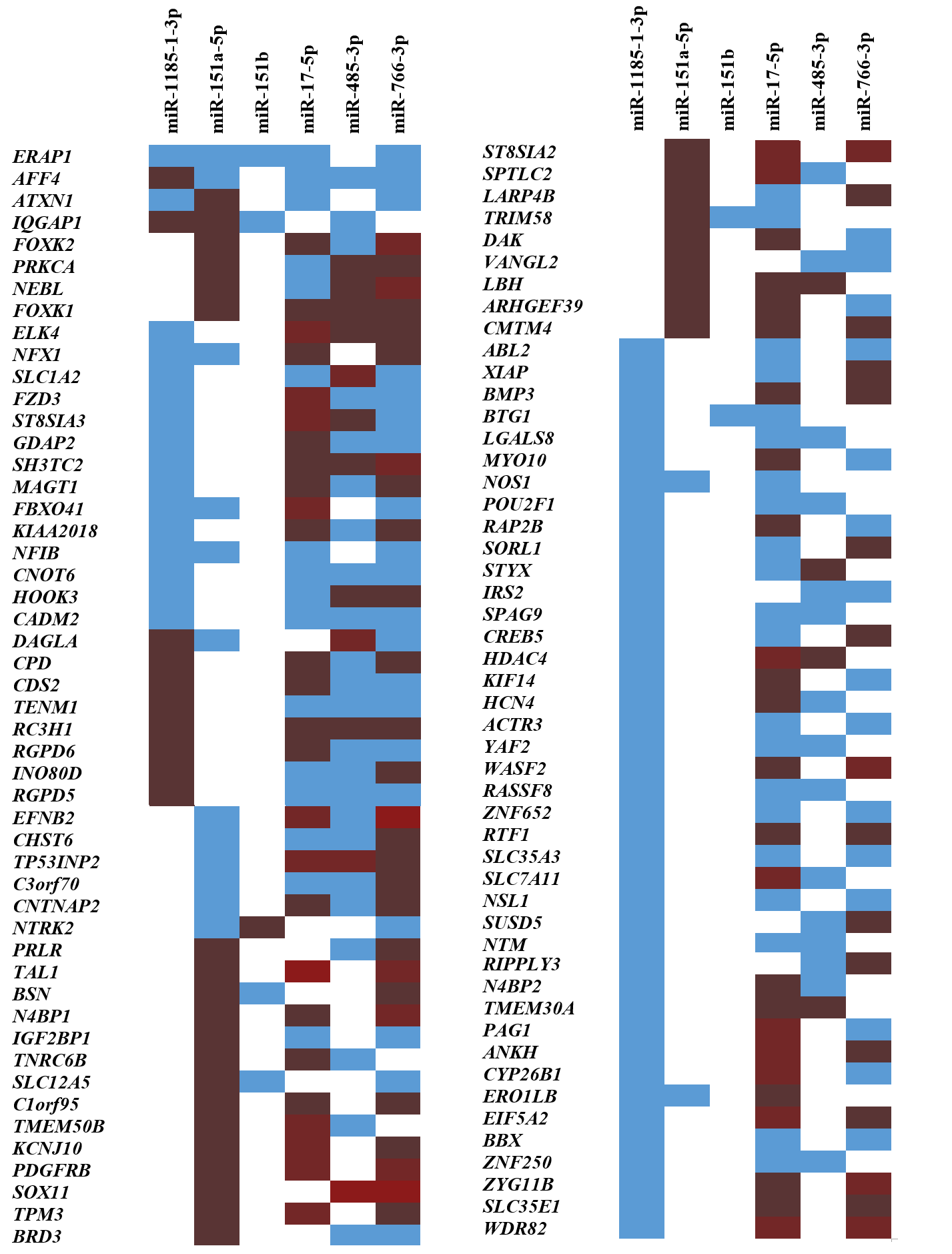
*


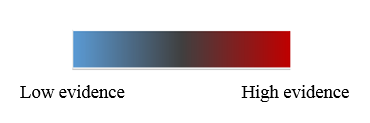


**Figure S3. Top 100 target genes predicted by downregulated miRNAs according to miRWalk 2.0.** The top genes are the targets predicted by more miRNAs and the color represents the evidence of this prediction.
